# SGLT2 Inhibitors Play a Salutary Role in Heart Failure via Modulation of the Mitochondrial Function

**DOI:** 10.3389/fcvm.2019.00186

**Published:** 2020-01-08

**Authors:** Yasuhiro Maejima

**Affiliations:** Department of Cardiovascular Medicine, Tokyo Medical and Dental University, Tokyo, Japan

**Keywords:** SGLT2, mitochondria, ketone body, NHE, fusion, fission

## Abstract

Three cardiovascular outcome trials of sodium glucose cotransporter 2 (SGLT2) inhibitors, including the EMPA-REG OUTCOME trial, CANVAS Program, and DECLARE TIMI 58 trial, revealed that SGLT2 inhibitors were superior to a matching placebo in reducing cardiovascular events, including mortality and hospitalization for heart failure, in patients with type 2 diabetes. However, the detailed mechanism underlying the beneficial effects that SGLT2 inhibitors exert on cardiovascular diseases remains to be elucidated. We herein review the latest findings of the salutary mechanisms of SGLT2 inhibitors in cardiomyocytes, especially focusing on their mitochondrial function-mediated beneficial effects. The administration of SGLT2 inhibitors leads to the elevation of plasma levels of ketone bodies, which are an efficient energy source in the failing heart, by promoting oxidation of the mitochondrial coenzyme Q couple and enhancing the free energy of cytosolic ATP hydrolysis. SGLT2 inhibitors also promote sodium metabolism-mediated cardioprotective effects. These compounds could reduce the intracellular sodium overload to improve mitochondrial energetics and oxidative defense in the heart through binding with NHE and/or SMIT1. Furthermore, SGLT2 inhibitors could modulate mitochondrial dynamics by regulating the fusion and fission of mitochondria. Together with ongoing large-scale clinical trials to evaluate the efficacy of SGLT2 inhibitors in patients with heart failure, intensive investigations regarding the mechanism through which SGLT2 inhibitors promote the restoration in cases of heart failure would lead to the establishment of these drugs as potent anti-heart failure drugs.

## Introduction

**Figure 1:**
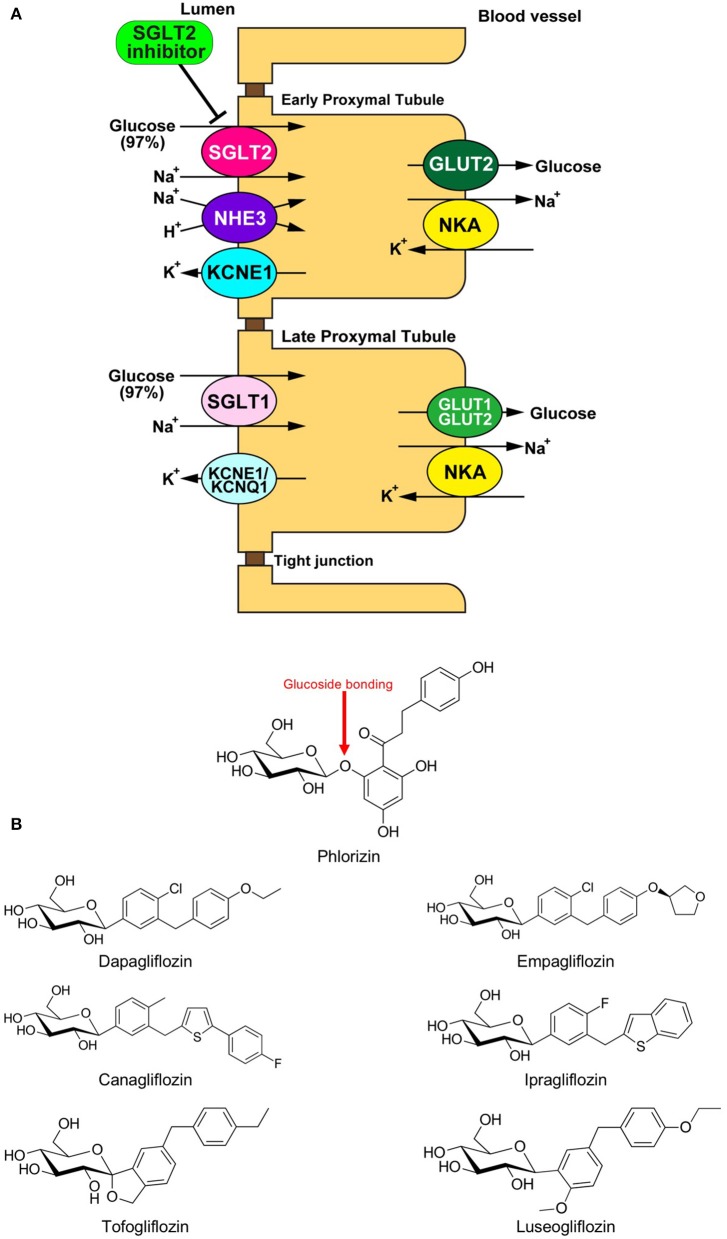
**(A)** Physiology of glucose reabsorption in the renal proximal tubules and the target of SGLT2 inhibitors. GLUT, glucose transporter; KCNE1, potassium voltage-gated channel Isk-related family member 1; KCNQ1, potassium voltage-gated channel KQT-like subfamily member 1; NHE, Na^+^/H^+^ exchanger; NKA, Na^+^/K^+^ ATPase; SGLT, sodium-dependent glucose transporter. **(B)** Chemical structural formulas of Phlorizin and SGLT2 inhibitors (Dapagliflozin, Empagliflozin, Canagliflozin, Ipragliflozin, Tofogliflozin, and Luseogliflozin).

Sodium glucose cotransporter (SGLT) is a channel protein that imports glucose into the intracellular space together with sodium ions (Na^+^) using the gradient of the Na^+^ concentration between inside and outside of the cells (Figure 1A) (1). SGLTs are expressed in limited organs, including the brain, small intestine, and renal tubule of mammals. Phlorizin, a phloretin that connects with glucose via glucoside bonding, is a natural compound derived from the bark of the apple tree root (Figure 1B). The administration of phlorizin leads to renal glycosuria, as this compound can inhibit SGLT1/2 located on the renal tubule, which results in the alleviation of hyperglycemia by discharging glucose to urine (Figure 1A) (2, 3). However, the intake of phlorizin causes severe diarrhea because this compound also inhibits small intestinal SGLT1, thereby suppressing the reabsorption of glucose together with water in the intestinal tract. To overcome this weakness of phlorizin, intensive analyses were conducted to investigate the molecular structures of both phlorizin and the SGLT receptor. Based on these analyses, highly selective SGLT2 inhibitors were developed as a novel type of anti-diabetes drug (Figure 1B) (4). In recent years, several cardiovascular outcome studies to test the safety of glucose-lowering drugs have demonstrated that SGLT2 inhibitors have a potential protective effect against cardiovascular events that is comparable to existing anti-heart failure drugs. However, it remains unknown how SGLT2 inhibitors exert such beneficial effects in patients with cardiovascular diseases. One of the major reasons why this has not been elucidated is that SGLT2 is not expressed in cardiomyocytes (5). Thus, it is largely believed that SGLT2 inhibitors play a protective role via the modulation of the internal environment outside of the myocardium (6). On the other hand, several investigators have shown that SGLT2 inhibitors directly manifest protective effects in the heart (6). In both cases, it is assumed that SGLT2 inhibitors exert their protective effects by restoring the mitochondrial function in cardiomyocytes. We herein review the current understanding on how SGLT2 inhibitors mitigate cardiac dysfunction through mitochondrial protection-mediated mechanisms.

## Clinical Evidence of the Cardioprotective Effects of SGLT2 Inhibitors

The EMPA-REG OUTCOME trial, a cardiovascular outcome trial (CVOT) of the SGLT2 inhibitor empagliflozin, demonstrated that empagliflozin was superior to a matching placebo in reducing cardiovascular events, including mortality and hospitalization for heart failure in patients with type 2 diabetes and established cardiovascular diseases (7, 8) (Table 1). The CANVAS Program, which consists of the CANVAS study and CANVAS-R, CVOTs assessed the cardiovascular safety and efficacy of the SGLT2 inhibitor canagliflozin in patients with type 2 diabetes and established cardiovascular disease, and also revealed that canagliflozin reduced the risk of a composite outcome of major adverse cardiovascular events in comparison to a matching placebo (9) (Table 1). Furthermore, the DECLARE TIMI 58 trial demonstrated that the SGLT2 inhibitor dapagliflozin reduced the risk of cardiovascular death or hospitalization for heart failure in comparison to a matching placebo in patients with type 2 diabetes and either a high cardiovascular risk or established atherosclerotic cardiovascular disease (10) (Table 1). As most patients in these trials did not have a diagnosis of heart failure at the time of study entry, the merit of treatment with an SGLT2 inhibitor largely reflected the prevention of heart failure development (11). Furthermore, the fact that reduction in the risk of hospitalization for heart failure emerged early after randomization raised the possibility that the mechanisms of the SGLT2 inhibitor-mediated cardiovascular benefits differ from those of existing glucose-lowering therapies that exert their effects independently of glycemic control. Indeed, a series of preclinical investigations demonstrated the effectiveness of SGLT2 inhibitors in animal models of non-diabetic heart failure. Byrne et al. revealed that the administration of empagliflozin alleviated left ventricular systolic dysfunction in non-diabetic mice subjected to pressure overload both *in vivo* and *ex vivo* (12). Andreadou et al. and Yurista et al. demonstrated that the administration of empagliflozin reduced the infarcted area of the myocardium, thereby improving the cardiac function in experimental non-diabetic myocardial infarction models (13, 14). In this background, randomized clinical trials were designed to explore the effects of SGLT2 inhibitors in patients with established heart failure with or without diabetes. Recently, the DAPA-HF trial demonstrated the significant advantage of dapagliflozin in reducing major adverse outcomes, such as unexpected hospitalization due to the exacerbation of heart failure, in patients with established heart failure with a reduced ejection fraction (HFrEF) (15). However, for SGLT2 inhibitors to be safely used for the treatment of non-diabetic heart failure, it is essential to elucidate their mechanism of action in detail. Thus far, a number of hypothesized mechanisms have proposed to explain the benefits of SGLT2 inhibitors in heart failure (6). Some investigators suggested that SGLT2 inhibitor-mediated natriuresis reduces the plasma volume or interstitial fluid, thereby favorably influencing ventricular remodeling by reducing the cardiac volume (16). Other investigators suggested that SGLT2 inhibitors alleviate heart failure through the suppression of sympathetic nervous activity, as evidenced by the reduction in arterial blood pressure without an increase in heart rate (7, 17). Still others hypothesized that SGLT2 inhibitors enhance the synthesis of erythropoietin by restoring the activity of “neural crest-derived” fibroblasts surrounding the renal proximal tubules, which, in turn, increases the delivery of oxygen to the failing myocardium (18). Thus, the targets through which SGLT2 inhibitors exert their protective effects against heart failure are mainly located outside of the heart. However, some investigations regarding this issue demonstrated that SGLT2 inhibitors have the potential to directly protect cardiomyocytes. Most such investigations have argued that SGLT2 inhibitors directly alleviate cardiac dysfunction through the modulation of mitochondria-associated mechanisms, including ketone body metabolism, sodium metabolism, and mitochondrial dynamics.

**Table 1:** Summary of cardiovascular outcome trials with SGLT2 inhibitors.

	**EMPA-REG Outcome**	**CANVAS Program**	**Declare-TIMI 58**
Study drug	Empagliflozin	Canagliflozin	Dapagliflozin
Drug class	SGLT2 inhibitor	SGLT2 inhibitor	SGLT2 inhibitor
Comparator	Placebo	Placebo	Placebo
Selected inclusion criteria	Adults with T2D at high risk of CV disease; BMI ≤ 45 kg/m^2^; no glucose-lowering therapy in previous 12 weeks and HbA1c 7.0–9.0%, or stable glucose-lowering therapy and HbA1c 7.0–10.0%	T2D; HbA1c 7.0–10.5%; age ≥30 years with a history of CV events, or age ≥50 years with a high risk of CV events; eGFR ≥30 ml/min/1.73 m^2^	T2D; HbA1c ≥6.5–
Selected exclusion criteria	ACS, stroke, or TIA in previous 2 months; planned cardiac surgery or angioplasty; liver disease; eGFR 2	T1D; diabetic ketoacidosis; pancreas or beta-cell transplantation; diabetes secondary to pancreatitis or pancreatectomy; severe hypoglycaemic episode in previous 6 months	T1D; CrCl
Number of patients	7,020	10,142	17,160
Study aim	Assess CV safety outcomes with empagliflozin compared with placebo, on top of standard of care, in patients with T2D at high CV risk	To pool results from the CANVAS and CANVAS-R trials to assess CV safety outcomes with canagliflozin compared with placebo, on top of standard of care, in patients with poorly controlled T2D and a history of CV events, or high risk of CV events	Assess CV outcomes with dapagliflozin compared with placebo, on top of standard of care, in patients with T2D who either have or are at risk of atherosclerotic CV disease
Primary outcome	3P-MACE (CV death, non-fatal MI or non-fatal stroke)	3P-MACE (CV death, non-fatal MI or non-fatal stroke)	Primary safety outcome: non-inferiority for 3P-MACE (CV death, non-fatal MI or non-fatal ischemic stroke). Co-primary efficacy outcomes: 3P-MACE; CV death or hospitalization for heart failure
Other key outcomes	4P-MACE (3P-MACE or hospitalization for unstable angina); CV death; hospitalization for heart failure; all-cause mortality; incident or worsening nephropathy	Individual components of composite endpoint; all-cause mortality; hospitalization for heart failure; progression of albuminuria	Composite kidney outcome (sustained ≥40% reduction in eGFR to 2, new ESKD, or kidney or CV death); all-cause mortality; hospitalization for heart failure
Number of events	772	1,011	–
Start date	2010-07-01	2014-01-01	2013-04-01
Median follow-up	3.1 years	CANVAS: ~5.7 years; CANVAS–R: ~2.1 years; CANVAS Program: ~2.4 years	4.2 years
Date of completion	2015-04-01	2017-02-01	2018-09-01
Key results	Primary outcome: HR 0.86 (95% CI 0.74, 0.99; *p* = 0.04 for superiority); 4P-MACE: HR 0.89 (95% CI 0.78, 1.01; *p* = 0.08 for superiority); CV death: HR 0.62 (95% CI 0.49, 0.77; *p* < 0.001) hospitalization for heart failure: HR 0.65 (95% CI 0.50, 0.85; *p* = 0.002); all-cause mortality: HR 0.68 (95% CI 0.57, 0.82; *p* < 0.001) incident or worsening nephropathy: HR 0.61 (95% CI 0.53, 0.70; *p* < 0.001)	CANVAS Program ITT analysis Primary outcome: 3P-MACE: HR 0.86 (95% CI 0.75, 0.97; *p* = 0.02 for superiority); all-cause mortality: HR 0.87 (95% CI 0.74, 1.01); CV death: HR 0.87 (95% CI 0.72, 1.06); hospitalization for HF: HR 0.67 (95% CI 0.52, 0.87); progression of albuminuria: HR 0.73 (95% CI 0.67, 0.79)	Co-primary efficacy outcomes−3P-MACE: HR 0.93 (95% CI 0.84, 1.03; *p* = 0.17 for superiority); CV death or hospitalization for heart failure: HR 0.83 (95% CI 0.73, 0.95; *p* = 0.005 for superiority); exploratory outcomes—kidney composite outcome: HR 0.76 (95% CI 0.67, 0.87); all-cause mortality: HR 0.93 (95% CI 0.82, 1.04); hospitalization for heart failure: HR 0.73 (95% CI 0.61, 0.88); CV death: HR 0.98 (95% CI 0.82, 1.17)
References	Zinman et al. *N Engl J Med* 2015; 373:2117; Wanner et al. *N Engl J Med* 2016; 375:323; NCT01131676	Neal et al. *N Engl J Med* 2017; 377:644; Neal et al. *Diabetes Obes Metab* 2017;19:926	Wiviott et al. *N Engl J Med* 2019; 380:347; NCT01730534
Sponsor	Boehringer Ingelheim & Eli Lilly and Company Diabetes Alliance	Janssen Research and Development, The George Institute for Global Health	AstraZeneca

## SGLT2 Inhibitors Increase the Amount of Ketone Bodies, Thereby Promoting Cardioprotective Effects

**Figure 2:**
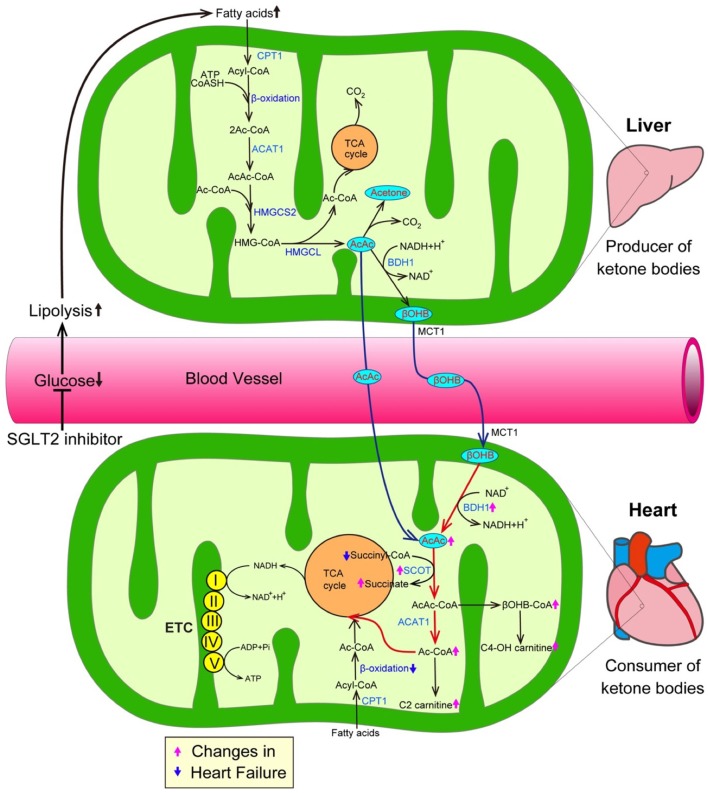
SGLT2 inhibitors increase the amount of ketone bodies, thereby promoting cardioprotective effects. The inhibition of SGLT2 reduces plasma glucose levels, thereby promoting lipolysis in adipose tissue, which in turn enhances the generation of ketone bodies. On the other hand, a growing body of evidence suggests that ketone bodies are favorable substrates in energy production because the conversion of ketone bodies to acetyl-CoA is much easier in comparison to the conversion of FFAs and glucose to acetyl-CoA. Furthermore, transcriptional level changes of ketone oxidation-related genes would be associated with the substrate shift to ketone bodies in the failing heart. Both pink and blue arrows show the changes in heart failure. AcAc CoA, Acetoacetyl CoA; ACAT1, Acetyl-CoA acetyltransferase; ADP, Adenosine diphosphate; ATP, Adenosine triphosphate; BDH1, Mitochondrial β-hydroxybutyrate dehydrogenase; βOHB, β-hydroxybutyrate; βOHB CoA, β-hydroxybutyryl CoA; C2-carnitine, Acetylcarnitine; C4-OH carnitine, Hydroxybutyrylcarnitine; CPT1, Carnitine palmitoyltransferase 1; ETC, Electron transport chain; HMGCL, 3-hydroxy-3-methylglutaryl-coenzyme A lyase; HMGCS2, 3-hydroxy-3-methylglutaryl-coenzyme A synthase 2; and SCOT, Succinyl-CoA:3-oxoacid-CoA transferase.

The inhibition of SGLT2 induces glucosuria, which thereby lowers plasma glucose levels, resulting in a reduction in the insulin level and an increase in the glucagon level during the fasting state. Such hormonal changes facilitate lipolysis in adipose tissue, and—at the same time—promote the conversion of carbohydrate to fat in whole-body substrate utilization. Thus, the administration of SGLT2 inhibitors could elevate ketone body levels in humans (Figure 2) (19). Ketone bodies, which are composed of acetoacetate (AcAc), β-hydroxybutyrate (βOHB), and acetone, are exclusively generated in the liver when the supply of glucose is impaired due to either a reduction of exogenous influx or deterioration of insulin signaling, or when the amount of free fatty acids (FFAs) is excessive due to the hyperactivation of lipolysis (20). During such situations, fatty acid β-oxidation is upregulated, thereby increasing the NADH/NAD^+^ ratio, which in turn promotes the conversion of AcAc to βOHB in the mitochondria of the liver (Figure 2). FFAs, a major source of ketone bodies, are taken up into hepatocytes, and β-oxidation transforms FFAs into acetyl-CoA and acetoacetyol-CoA (AcAc-CoA). 3-hydroxy-3-methylglutaryl-coenzyme A synthase 2 (HMGCS2), a rate-limiting mitochondrial enzyme, catalyzes the condensation of acetyl-CoA and AcAc-CoA to generate 3-hydroxy-3-methtylglutaryl-CoA (HMG-CoA) (21). Subsequently, 3-hydroxy-3-methylglutaryl-coenzyme A lyase (HMGCL) sequentially cleaves HMG-CoA into acetyl-CoA and AcAc (22, 23). Then, D-β-hydroxybutyrate dehydrogenase (BDH1) converts AcAc to βOHB, a more stable form of ketone body. In addition, both the kidneys and intestines play a critical role in maintaining ketone body homeostasis by regulating the ketone-reabsorptive capacity through sodium-dependent monocarboxylate transporter (SMCT) 1 and 2. Ketone bodies diffuse into the circulation and are used as an energy source in various organs (24). In the mitochondria of the heart, ketone bodies are rapidly converted to acetyl CoA through catalyzation with several enzymes, such as βOHB dehydrogenase (BDH1), succinyl-CoA:3-oxoacid-CoA transferase (SCOT), and mitochondrial acetyl-CoA acetyltransferase 1 (25).

The mammalian heart requires a vast amount of energy to maintain a normal contractile function and intracellular energy storage is limited. Thus, cardiomyocytes must generate an enormous amount of adenosine triphosphate (ATP) via the oxidation of carbon fuel. Under normal conditions, the predominant energy source of cardiomyocytes is FFAs, which provide 60% of the myocardial ATP demand by β-oxidation (26). The remaining 40% of the myocardial ATP demand is provided by carbohydrate oxidation, including glycolysis. The proportions of the energy sources of cardiomyocytes dynamically changes according to conditions such as exercise, feeding and starvation. When the mitochondrial oxidative metabolism balance of cardiomyocytes is seriously damaged due to various stresses including hypoxia and pressure overload, the major origin of ATP shifts from β-oxidation-mediated FA degradation to carbohydrate oxidation-mediated glucose catabolism. Such metabolic adaptation during hypoxia is reasonable because the glycolysis pathway can work, even under anaerobic conditions. However, as the efficiency of ATP production in glycolysis is significantly lower than that in mitochondrial oxidative metabolism, more efficient energy sources are required in the failing heart, in which the oxygen supply is impaired for an extended period of time (27). From this perspective, ketone bodies are a favorable substrate for energy production because the conversion of ketone bodies to acetyl-CoA is much easier in comparison to the conversion of FFAs and glucose (Figure 2) (28, 29). More importantly, ketone bodies can lead to the more efficient oxidation of the mitochondrial coenzyme Q couple and enhance the free energy of cytosolic ATP hydrolysis. Furthermore, changes at the transcriptional level of ketone oxidation-related genes would be associated with the substrate shift to ketone bodies in the failing heart. Indeed, previous investigations revealed that ketone metabolism is increased accompanied with the decrease of fatty acid oxidation in failing heart, as evidenced by the elevation of the levels of BDH1 and ketone body-derived materials, such as hydroxybutyryl-carnitine (C4OH-carnitine), βOHB-CoA and acetyl-carnitine (C2-carnitine) (28, 29) (Figure 2). In addition, a number of studies have demonstrated that the intake of a ketogenic diet extends longevity and the health span (30). Shimazu et al. revealed part of its mechanism. Treatment with βOHB inhibits histone deacetylase, thereby promoting FoxO3A and MT2 activity, which, in turn, markedly reduce oxidative stress and extend the life span in mice (31). Furthermore, ketone bodies possess anti-inflammatory activity (32). Youm et al. demonstrated that ketone bodies play an anti-inflammatory role by inhibiting the activity of the NOD-like receptor pyrin domain containing protein 3 (NLRP3) inflammasomes in animal models (33).

Thus, the elevation of ketone levels by SGLT2 inhibition might have a beneficial effect in patients with heart failure through multiple mechanisms.

## SGLT2 Inhibitors Promote Sodium Metabolism-Mediated Cardioprotective Effects

**Figure 3:**
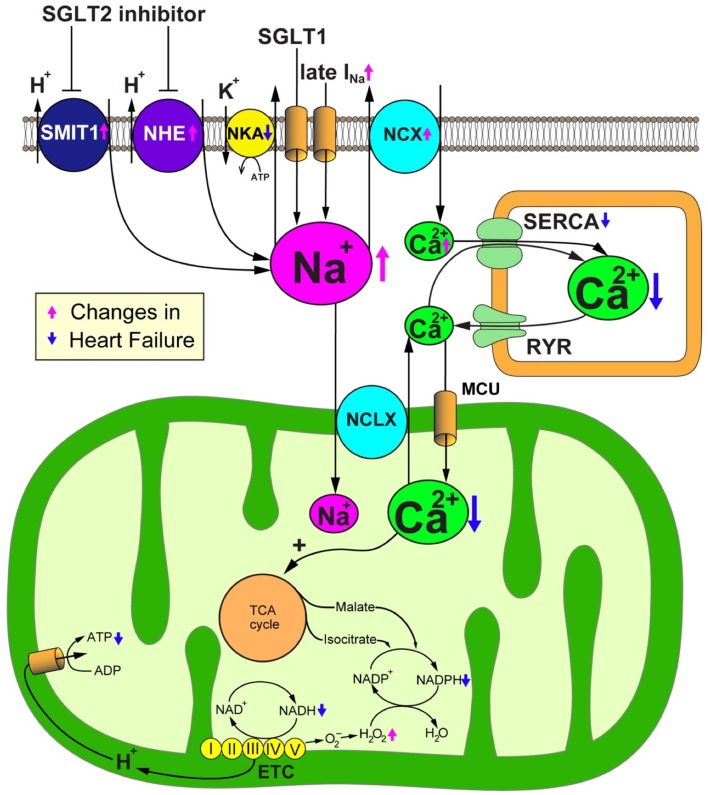
SGLT2 inhibitors promote sodium metabolism-mediated cardioprotective effects. Failing cardiomyocytes show elevated intracellular Na^+^ concentrations due to (1) increased Na^+^ influx via the late Na^+^ current (I_Na_), (2) enhanced sarcolemmal Na^+^/H^+^ exchanger (NHE) activity, (3) reduced Na^+^/K^+^ ATPase (NKA) activity, and in the case of the diabetic heart, (4) the increased expression and activity of Na^+^-glucose cotransporter 1 (SGLT1). Intracellular overload of Na^+^ promotes Ca^2+^ efflux from mitochondria through the mitochondrial Na^+^/Ca^2+^ exchanger (NCLX). The reduction of the Ca^2+^ concentration in the mitochondrial matrix deteriorates the Ca^2+^-induced upregulation of TCA cycle dehydrogenases in response to workload transition, thereby disturbing the regeneration of reducing equivalents that are essential for preserving the antioxidative capacity and matching the energy supply to the energy demand. SGLT2 inhibitors would have a salutary role in failing cardiomyocytes through their alleviation of Na^+^ and Ca^2+^ handling through NHE inhibition. ADP, adenosine diphosphate; ATP, adenosine triphosphate; ETC, electron transport chain; MCU, mitochondrial Ca^2+^ uniporter; NAD^+^/NADH, nicotine amide dinucleotide oxidized/reduced; NCX, sarcolemmal Na^+^/Ca^2+^ exchanger; NKA, Na^+^/K^+^ ATPase; RyR, ryanodine receptor; SERCA, sarcoplasmic reticulum Ca^2+^ ATPase.

As the inhibition of SGLT2 induces natriuresis as well as glycosuria because SGLT2 cotransports glucose with sodium, SGLT2 blockade could alter intracellular sodium homeostasis. Sodium plays an important role in mitochondrial redox regulation and excitation-contraction coupling in cardiomyocytes (Figure 3) (34, 35). Indeed, to produce energy in the form of ATP, cardiomyocytes primarily depend on the mitochondrial oxidative phosphorylation system (OXPHOS). Nicotinamide adenine dinucleotide (NADH), a reducing equivalent that is produced from the tricarboxylic acid (TCA) cycle, donates its electron to complexes I, III and IV of the electron transport chain (ETC), thereby promoting the translocation of H^+^ to the mitochondrial intermembrane space. The reduced form of flavin adenine dinucleotide (FADH_2_) also participates in the ETC reaction by donating its electron to complex II. The concentration gradient of H^+^ translocation generated by this reaction drives the conversion from ADP to ATP at the F1/F0-ATP synthase. The increase of ADP caused by the increased energy demand enhances the production of ATP at the F1/F0-ATP synthase, thereby promoting the oxidization of NADH to NAD^+^. Concurrently, the increase of cytosolic Ca^2+^ transients by β-adrenergic stimulation promotes the uptake of mitochondrial Ca^2+^ through the mitochondrial Ca^2+^ uniporter (MCU) (36). Then, Ca^2+^ activates the dehydrogenases of the TCA cycle to promote the regeneration of NADH (37). Thus, OXPHOS acts in concert with the TCA cycle to preserve constant ratios of ATP/ADP and NADH/NAD^+^ (38). In addition, nicotinamide adenine dinucleotide phosphate (NADPH) which is produced from NADH and TCA cycle products such as malate and isocitrate, plays a critical role in maintaining oxidative defense by donating electrons to reduced glutathione, thioredoxin, and glutaredoxin pools. Thus, the mitochondrial Ca^2+^ uptake is crucial for preserving the mitochondrial antioxidative capacity as well as for matching the energy supply to the demand (39). Ca^2+^ handling in cardiomyocytes is closely coordinated with Na^+^ handling through the activity of the sarcolemmal Na^+^/Ca^2+^ exchanger (NCX) and the mitochondrial Na^+^/Ca^2+^ exchanger (NCLX). The cardiac NCX entirely bails out Ca^2+^ to the extracellular space under physiological conditions. However, NCX sets out to import Ca^2+^ to the cytosol in the early phase of the action potential, depending on the membrane potential and the Na^+^ and Ca^2+^ transmembrane gradients (40). The cardiac NCLX is mainly responsible for the extrusion of Ca^2+^ from mitochondria. However, as the kinetics of NCLX are slower in comparison to the uptake of Ca^2+^ via the MCU, it is susceptible to the accumulation of Ca^2+^ in mitochondria after increasing the rate and amplitude of cytosolic Ca^2+^ transients.

Heart failure is closely associated with the impairment of both Ca^2+^ and Na^+^ handling in cardiomyocytes. Indeed, the amplitude and velocity of cytosolic Ca^2+^ transients are decreased in failing cardiomyocytes. Furthermore, the elevation of diastolic cytosolic Ca^2+^ ([Ca^2+^]_c_) and Na^+^ concentrations ([Na^+^]_c_) is observed in failing cardiomyocytes (41, 42). The impairment of Ca^2+^ handling is due to the decrease of the Ca^2+^ uptake by the sarco/endoplasmic reticulum Ca^2+^- ATPase (SERCA) and the leak of Ca^2+^ from the sarcoplasmic reticulum (SR) via ryanodine receptors (43, 44). The increase in the expression and activity of the NCX promotes the export of Ca^2+^ into the extracellular space, and thereby also reduces the Ca^2+^ load of the SR (45). Furthermore, the reduction of the release of Ca^2+^ from the SR results in the impairment of the mitochondrial Ca^2+^ uptake and steady-state Ca^2+^ concentration ([Ca^2+^]_m_) (46). On the other hand, excessive influx of Ca^2+^ into the mitochondria is detrimental to cardiomyocytes. The elevation of [Ca^2+^]_m_ triggers depolarization of mitochondrial inner membrane potential, generation of reactive oxygen species (ROS), and opening the mitochondrial permeability transition pore (47, 48), thereby promoting the release of pro-apoptotic proteins, such as cytochrome c, into the cytosol (49).

Increasing lines of evidence suggest that [Na^+^]_c_ is significantly elevated in failing cardiomyocytes as a result of (1) increased Na^+^ influx via the late Na^+^ current (I_Na_) (41), (2) enhancement of sarcolemmal Na^+^/H^+^ exchanger (NHE) activity (50), (3) reduction of Na^+^/K^+^ ATPase (NKA) activity (51), and—in the case of diabetic heart—(4) the increased expression and activity of the Na^+^-glucose cotransporter 1 (SGLT1) (52) (Figure 2). Generally, the increase of [Na^+^]c should trigger positive effects on cytosolic Ca^2+^ handling because intracellular Na^+^ overload prevents the NCX from exporting Ca^2+^ during the diastolic phase and promotes the reverse-mode function of the NCX during the action potential—thereby enhancing additional trans-sarcolemmal Ca^2+^ influx to achieve the elevation of Ca^2+^ in the SR and increase the amplitude of cytosolic Ca^2+^ transients. However, from a metabolic point of view, the elevation of [Na^+^]_c_ results in detrimental effects, especially in mitochondria. As Ca^2+^ is pumped out of mitochondria to the cytosol by an NCLX, the elevation of [Na^+^]c enhances the driving force for mitochondrial Ca^2+^ efflux. The decrease of [Ca^2+^]_m_ suppresses the Ca^2+^-induced upregulation of dehydrogenases in the TCA cycle, thereby attenuating the production of both NADH and NADPH (46). The decreased production of NADH causes ATP depletion. The reduction of the amount of NADPH causes the impairment of mitochondrial antioxidative defense because the donation of electrons from NADPH is indispensable for antioxidative enzymes, such as peroxiredoxin, glutathione peroxidase, and glutaredoxin. Thus, the elevation of [Na^+^]_c_ enhances oxidative stress, thereby aggravating the vulnerability of the heart to arrhythmias and neurohormonal hyperactivation. Furthermore, the increase of [Na^+^]_c_ eventually causes the emission of mitochondrial ROS, which results in the further deterioration of the intracellular Na^+^ overload (35). Based on these facts, reducing the intracellular Na^+^ overload to improve mitochondrial energetics and oxidative defense could be a promising therapeutic strategy for heart failure (Figure 3).

With regard to the beneficial effects of SGLT2 inhibitors on heart failure, it was initially considered that SGLT2 inhibitors have no direct effect on cardiomyocytes because SGLT2 is not expressed in the heart in either healthy subjects or under pathological conditions (5). However, a recent investigation demonstrated that empagliflozin reduced [Na^+^]_c_ and [Ca^2+^]_c_ in isolated cardiomyocytes (53). According to this report, empagliflozin directly reduced myocardial [Na^+^]_c_ and [Ca^2+^]_c_ and elevated [Ca^2+^]_m_ by suppressing myocardial NHE flux, independently of glucose transport. Habibi et al. demonstrated that the administration of empagliflozin mitigates diastolic dysfunction in db/db mice (54). The author of the present study found that empagliflozin suppresses the expression of serum- and glucocorticoid-inducible kinase 1 (SGK1) in the myocardium. As SGK1 activity may modulate NHE activity through Akt-mediated signaling, these results suggest that empagliflozin could restore myocardial [Na^+^]_c_ in a sustained manner (55). Examinations using ^23^Na^+^ magnetic resonance imaging revealed that the tissue Na^+^ content in diabetic patients was markedly reduced by treatment with dapagliflozin (56). An *in silico* docking study demonstrated that three SGLT2 inhibitors, empagliflozin, dapagliflozin, and canagliflozin, showed high binding affinity with the extracellular Na^+^-binding site of NHE (57). In this study, the authors confirmed—by *in vitro* experiments—that empagliflozin, dapagliflozin and canagliflozin directly inhibit the cardiac NHE flux and reduce [Na^+^]_c_.

The expression of NHE is upregulated in the failing heart, possibly through the acidification of the intracellular environment due to increased conversion of pyruvate to lactate (58). Similarly, the NHE activity of cardiomyocytes of the animal models of type 2 diabetes and the suppression of [Na^+^]_c_ by NHE inhibition with cariproride was found to be cardioprotective (59, 60). Specifically, cariproride significantly suppressed the elevation of [Na^+^]_c_ at the end of ischemia and inhibited ventricular arrhythmia during reperfusion in a db/db mouse model of ischemia/reperfusion (59). In the Goto–Kakizaki rat model of type 2 diabetes, which does not develop hypertension, obesity or hyperlipidemia, the NHE activity of cardiomyocytes is markedly upregulated, which results in an increase in [Na^+^]_c_. In this model, the intracellular Na^+^ overload was closely associated with the Akt-mediated progression of left ventricular hypertrophy. Consistently, the administration of cariproride significantly suppressed both [Na^+^]_c_ and Akt activation, resulting in the attenuation of cardiac hypertrophy (60).

There are seven SGLT isoforms (SGLT1 to 6 and sodium-myoinositol cotransporter 1, SMIT1). Among these, only SGLT1 and SMIT1 are expressed in the mammalian heart. The overexpression of SMIT1 activates NOX2, increases ROS, and exacerbates glucotoxicity in cardiomyocytes. Consistently, the deletion of SMIT1 prevented hyperglycemia-induced NOX2 activation (61). Thus far, the physiological role of SMIT1 in the heart remains unknown, as the deletion of SMIT1 does not alter the cardiac phenotype. Interestingly, however, SMIT1 is hardly associated with the glucose uptake in the heart, regardless of any glycemic conditions. Thus, SMIT1-mediated NOX2 activation would modulate glucose sensitization, which could trigger ionic signaling ([Na^+^]_c_ and [Ca^2+^]_c_ via the NCX) into cells in association with the changes in the extracellular glucose concentration. Concomitantly, intracellular signaling via protein kinase C (PKC)-β, a calcium-dependent serine/threonine kinase, could be the link to ionic changes downstream of SMIT1. The IC_50_ of empagliflozin and canagliflozin for SMIT1 are estimated to be 8.3 and 5.6 μM, respectively (62, 63). Indeed, empagliflozin is even able to reduce [Na^+^]_c_ in the absence of glucose (53).

## SGLT2 Inhibitors Could Modulate Mitochondrial Dynamics Resulting in Cardioprotection

**Figure 4:**
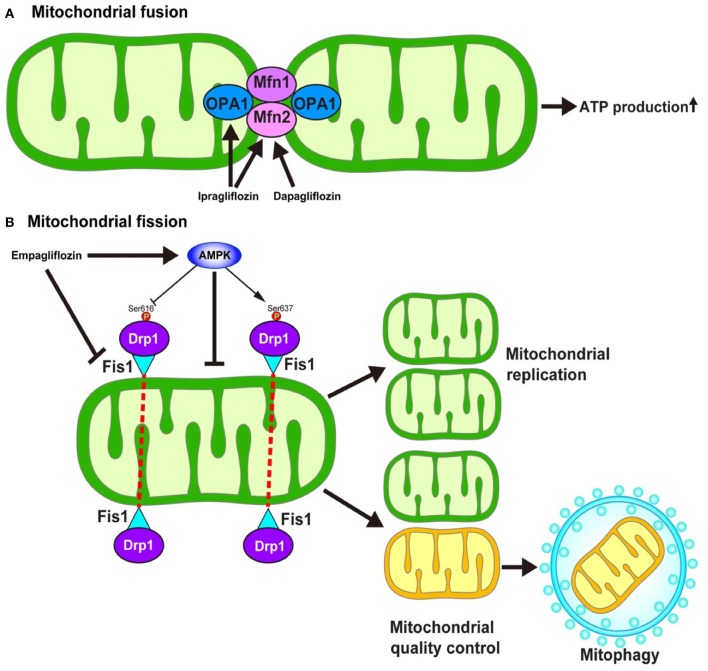
Hypothesized mechanism of the modulation of mitochondrial dynamics by SGLT2 inhibitors. The inhibition of SGLT2 might be associated with the mitochondrial dynamics through the regulation of **(A)** mitochondrial fusion and **(B)** mitochondrial fission. However, the detailed mechanism as to how SGLT2 inhibitors modulate the regulators of mitochondrial dynamics is largely unknown. AMPK, AMP-activated protein kinase; Drp1, Dynamin-related protein 1; Fis1, Mitochondrial fission 1 protein; Mfn, Mitofusin; Ser, Serine.

Mitochondria continuously fuse and divide in highly regulated manners to maintain their functions, which include metabolism, energy production, intracellular signaling, and the regulation of apoptosis. The enhancement of mitochondrial fusion would allow for the making up of “healthy” mitochondria, resulting in the normalization of the overall mitochondrial function. In response to various stresses, mitochondria undergo stress-induced mitochondrial hyperfusion (64), which thereby enhances ATP production, which—in turn—plays a pro-survival role. On the other hand, damaged mitochondria must be removed to preserve mitochondrial homeostasis. To this end, mitochondrial fission could be enhanced to more easily remove dysfunctional mitochondria via mitochondria-selective autophagy, termed mitophagy (65). Several key regulators are required for the operation of such mitochondrial dynamics. Mitochondrial fusion is regulated by mitofusin1 (Mfn1), mitofusin2 (Mfn2), and Opa1 (Figure 4) (66). On the other hand, mitochondrial fission is regulated by the recruitment of Dynamin-related protein 1 (Drp1) to specific sites on the outer mitochondrial membrane in coordination with mitochondrial fission 1 (Fis1) and mitochondrial fission factor (Mff) (Figure 4) (67).

The impairment of mitochondrial fusion via the downregulation of Mfn1 and Mfn2 aggravates cardiac dysfunction both at baseline and in response to stress (68). On the other hand, the inhibition of mitochondrial fission by the pharmacological suppression of Drp1 with Mdivi-1 reduces the size of infarcts that develop in response to ischemia/reperfusion (I/R) (69). In contrast, the inhibition of mitochondrial fission by genetic modulation, such as the knockdown of Fis1 mRNA or the expression of dominant-negative mutation in Drp1, inhibits mitophagy which results in metabolic dysfunction in INS1 cells (70), suggesting that mitochondrial fission has a two-sided nature with respect to cell survival in the myocardium.

Increasing lines of evidence suggest that SGLT2 inhibitors may modulate mitochondrial dynamics. Ipragliflozin alleviates the mitochondrial dysfunction induced by a high-fat diet by restoring the levels of Opa1 and Mfn2 to normal values *in vivo* without reducing body weight or blood glucose levels in rat models (71). Similarly, dapagliflozin normalizes the Mfn1/Mfn2 ratio in the rat model of metabolic syndrome, thereby suppressing prolonged ventricular repolarization (72). Empagliflozin restores the AMP/ATP ratio, thereby activating adenosine monophosphate (AMP)-activated protein kinase (AMPK) (73). The activation of AMPK causes an increase in Drp1^S637^ phosphorylation and a decrease in Drp1^S616^ phosphorylation, which results in the suppression of mitochondrial fission. Another study demonstrated that empagliflozin normalized the size and number of mitochondria in the OLETF diabetic rat heart and that the diabetes-induced excessive reduction in mitochondrial size after MI was inhibited by empagliflozin via the suppression of Fis1 upregulation and following ROS production, which results in the reduction of the MI size (74).

Thus, inhibition of SGLT2 is closely associated with the mitochondrial dynamics through the regulation of fusion and fission of mitochondria. Although several hypotheses have been proposed (71, 74, 75), the detailed molecular mechanism through which mitochondrial fusion and fission are modulated by the administration of SGLT2 inhibitors is largely unknown. Furthermore, it remains to be determined whether the effect of SGLT2 inhibitors on AMPK activity, one of the key molecules in the regulation of mitochondrial fission, is a class effect or a drug-specific effect. Indeed, Mancini et al. reported that canagliflozin, but not dapagliflozin or empagliflozin, could enhance AMPK activity both in human umbilical vein endothelial cells and human arterial endothelial cells (76). In addition, the precise roles of mitochondrial fission and fusion in the development of heart failure remain to be determined.

## Future Directions

We reviewed the proposed cardioprotective effect of SGLT2 inhibitors, which is mediated through the improvement of the mitochondrial function by (1) increasing ketone body usage, (2) the mitigation of sodium metabolism, and (3) the modulation mitochondrial dynamics. However, many questions remain to be solved to validate these hypotheses. Indeed, it remains controversial whether SGLT2 inhibitors could be directly involved in the protective effects of cardiomyocytes, which do not express SGLT2. In particular, regarding the regulation of mitochondrial dynamics, previous studies merely observed the change in the expression levels of factors that regulate the mitochondrial dynamics (e.g., Mfn1 or Drp1) in response to the administration of SGLT2 inhibitors. Thus, the molecular mechanism through which these compounds modulate mitochondrial fusion and fission remains to be elucidated. Regarding the association with ketone body metabolism, it is necessary to determine whether the favorable effects induced by the increase in ketone bodies would be limited in the alteration of the mitochondrial energy metabolism. Furthermore, the possibility that these drugs could regulate different target molecule(s) other than SGLT2 (i.e., have off-target effects) should be examined. Indeed, the hypothesis that SGLT2 inhibitors regulate sodium metabolism is based on the fact that SGLT2 inhibitors possess the potential to inhibit both NHE and SMIT1.

As stated above, the DAPA-HF trial demonstrated that dapagliflozin plays a protective role in patients with established HFrEF, regardless of the presence of diabetes (15). Currently, the EMPEROR-Reduced trial [NCT03057977] to evaluate the efficacy of empagliflozin vs. placebo on top of guideline-directed medical therapy in HFrEF patients with or without diabetes is ongoing (77). If empagliflozin is proven to be beneficial in patients with HFrEF based on the results of this trial, it would provide more robust evidence of the beneficial effect of SGLT2 inhibitors on heart failure. At the same time, two randomized clinical trials are evaluating the effects of SGLT2 inhibitors in patients with established heart failure with a preserved ejection fraction (HFpEF), regardless the presence of diabetes. One is the EMPEROR-Preserved trial [NCT03057951] with empagliflozin (78), and the other one is the DELIVER trial [NCT03619213] with dapagliflozin. Several preclinical studies proposed the mechanism how SGLT2 inhibitor alleviates cardiac diastolic dysfunction, a major cause of HFpEF. For example, Juni et al. demonstrated that Empagliflozin suppresses TNF-α-induced mitochondrial and cytoplasmic ROS accumulation, thereby restoring cardiac microvascular endothelial cell-derived NO delivery, which in turn leads to reinstatement of cardiac relaxation and contraction (79). There are great expectations regarding the result of these clinical trials because, at the time of writing, no drugs have been demonstrated to be effective for the treatment of HFpEF (80).

As is the case with the positive effects, unfavorable aspects of SGLT2 inhibitor administration for the heart failure patients should be considered. Increasing lines of evidence suggest that sarcopenia is one of the major risk factors for morbidity and mortality of heart failure. Past clinical observations demonstrated that the skeletal muscle mass reduction is observed in a given number of patients with diabetes who were treated with SGLT2 inhibitors. Also, the decreased exercise capacity, one of the major causes of sarcopenia which is the consequence of mitochondrial dysfunction in skeletal muscles, is an independent predictor of the poor prognosis of patients with heart failure (81). Thus, basically, the patients who are susceptible to sarcopenia should not be prescribed SGLT2 inhibitors. On the other hand, a recent investigation demonstrated the intriguing result that Empagliflozin restored decreased exercise endurance capacity by alleviating skeletal muscle fatty acid oxidation in an animal heart failure model (82). In any case, we should carefully determine which kind of patients are optimal for the treatment with SGLT2 inhibitors.

Taken together, unremitting efforts to elucidate the molecular mechanism through which the administration of SGLT2 inhibitors alleviates heart failure, as well as clinical studies of these compounds for non-diabetic heart failure could shift their classification from merely anti-diabetic drugs to potent anti-heart failure drugs.

## Author Contributions

The author confirms being the sole contributor of this work and has approved it for publication.

### Conflict of Interest

The author declares that the research was conducted in the absence of any commercial or financial relationships that could be construed as a potential conflict of interest.
